# A novel methodology to identify and survey physicians participating in medical aid-in-dying

**DOI:** 10.1038/s41598-022-09971-7

**Published:** 2022-04-11

**Authors:** Vinay Kini, Bridget Mosley, Julie Ressalam, Dragana Bolcic-Jankovic, Hillary D. Lum, Elizabeth R. Kessler, Matthew DeCamp, Eric G. Campbell

**Affiliations:** 1 Division of Cardiology, Weill Cornell Medical College, 520 E 70th St, Starr 433, New York, NY 10021 USA; 2 Department of Family Medicine, University of Colorado Anschutz Medical Campus, Aurora, USA; 3 Center for Bioethics and Humanities, University of Colorado Anschutz Medical Campus, Aurora, USA; 4 Center for Survey Research, University of Massachusetts Boston, Boston, USA; 5 Division of Geriatric Medicine, University of Colorado Anschutz Medical Campus, Aurora, USA; 6 Division of Medical Oncology, University of Colorado Anschutz Medical Campus, Aurora, USA; 7 General Internal Medicine, University of Colorado Anschutz Medical Campus, Aurora, USA

**Keywords:** Medical ethics, Health policy

## Abstract

Physicians who participate in medical-aid-in-dying (MAID) cannot be easily identified and studied due to cost and anonymity barriers. We developed and empirically tested a novel methodology to identify and survey physicians highly likely to participate in MAID activities. We used a state-level comprehensive administrative claims database to identify a cohort of patients with diagnoses and hospice enrollment similar to those known to have filled a prescription for MAID from 2017–2018. We then identified physicians who provided routine outpatient care to these patients using National Provider Identifier numbers. We surveyed these physicians in 3 waves (n = 583 total surveys), ranking physicians in order of their likelihood of being asked about MAID for each wave based on characteristics including specialty and the number of unique patients they had provided care to. We re-ranked physicians in waves 2 and 3 based on responses from prior waves. Physicians were surveyed only once and there was no follow-up to preserve anonymity. Surveys assessed the proportion of respondents who participated in MAID activities (discussions, referrals, and/or prescriptions). We identified 6369 physicians that provided care to 2960 patients. In survey waves one, two, and three respectively, response rates (55%, 52%, and 55%; p = 0.98) and the proportion of respondents that participated in MAID activities (58%, 56%, and 42%; p = 0.05) were similar. Small adjustments made to physician ranking criteria in waves two and three did not increase the proportion of physicians that participated in MAID activities. We used a novel methodology using administrative data to identify and survey physicians at high likelihood of participating in MAID activities. We achieved good overall response rates (52%), and a high proportion of respondents that participated in MAID activities (52%), demonstrating that it is possible to overcome cost and anonymity barriers to conducting quantitative research on MAID. This methodology could be used in larger scale studies of MAID or other bioethical issues with “hidden” physician populations.

## Introduction

Medical aid-in-dying (MAID), or the ability of physicians to prescribe medications to allow terminally ill patients to die when and where they chose, is expanding in the U.S. Nearly 20% of the U.S. population currently live in areas where MAID is legal, and the eligible population is likely to increase over time due to increased MAID legalization^1^. Despite the expansion of MAID, little is known about the characteristics or experiences of physicians participating in MAID, because physicians participating in MAID are a “hidden population”^2^. These physicians cannot be systematically identified to participate in scientific studies for several reasons: (1) states, employers and physician groups are charged with protecting physician confidentiality and will not release the identities of physicians participating in MAID, (2) pharmacies are legally prohibited from identifying physicians who write MAID prescriptions, (3) MAID prescriptions or visits often are not covered by insurance, and thus not directly available from claims databases, and (4) death certificates do not include information on whether MAID was used by statute.

Prior survey studies of MAID have used broad, large samples of physicians to identify a small number of physicians who have participated in MAID^3–6^. For example, Ganzini et al. surveyed 4500 physicians in Oregon and found 144 who had received a request for a MAiD prescription^3^. Today these large scale surveys are often cost prohibitive in terms of money and time^7^. Efforts to improve the efficiency of these surveys, such as sampling physicians that have specialties with higher likelihood of MAID participation (e.g., oncology or psychiatry) have shown only modest success and reduce the generalizability of the findings^8–10^.

Accordingly in this study, we developed and empirically tested a novel methodology using a state level all-payer administrative claims database to (1) identify patients in the state of Colorado that had characteristics similar to those that were prescribed MAID, and (2) identify the group of physicians that provided routine care to these patients. We hypothesized that our targeted sampling method would produce a higher proportion of respondents participating in MAID activities, and at lower cost, compared to prior surveys that did not use targeted sampling techniques.

## Methods

### Data sources

We used two data sources. To identify patients with terminal illnesses and the physicians who provided outpatient care to them, we used the Colorado All-Payer Claims Database (APCD). The APCD is a statewide, comprehensive administrative dataset that includes inpatient, outpatient, physician, and facility claims on nearly all patients who receive care in Colorado. Reporting is required for all insurance companies and plans with the exception of federal health facilities (e.g., Veterans Health Administration hospitals) and self-insured group health plans. Thus, it is not a voluntary effort that could lead to reporting bias. The APCD also includes beneficiary demographics including age and sex, insurance carrier, and hospital identifiers, but does not include reliable data on race/ethnicity. These data are available for purchase by researchers. To obtain additional information on physician characteristics, we used commercially available data from IQVIA. These data link National Provider Identifiers (NPIs, available from the APCD) with information such as physician specialty, practice location, and mailing address.

### Creation of patient cohort

We sought to identify a cohort of patients similar to the patients known to have filled a MAID prescription in Colorado in 2017–2018. Publicly available reports from the Colorado Department of Public Health and Environment (CDPHE) show that of the 193 patients who filled a MAID prescription in 2017–2018, the most common conditions were malignant neoplasms, progressive neurodegenerative diseases (such as amyotrophic lateral sclerosis and progressive supranuclear palsy), chronic lower respiratory diseases (such as chronic obstructive pulmonary disease) and heart diseases (such as heart failure)^11^. The CDPHE also reports that greater than 75% of the patients who received a MAID prescription were enrolled in hospice. Thus, our patient cohort inclusion criteria included (1) a diagnosis listed above and (2) receipt of hospice services.

We used ICD-10 (International Classification of Diseases, Tenth Revision) codes to identify patients with these diagnoses, and Current Procedural Technology (CPT) and Healthcare Common Procedure Coding System (HCPCS) codes to identify patients that received hospice services (Supplemental Table). Among all patients who received any healthcare services in Colorado from 2017–2018, we identified those with ICD-10 primary diagnosis codes for any inpatient or two outpatient claims in 2017–2018 for the conditions listed in the CDPHE report. Among these patients, we then identified those who received hospice services using CPT and HCPCS codes for hospice care planning or modifiers for services provided by hospice care.

### Creation of physician cohort

We then identified a cohort of physicians who provided routine, outpatient care to the patient cohort described above. We hypothesized that opportunities for patients to ask about MAID would be most common during outpatient clinic visits. Therefore, among the cohort of patients described above, we identified outpatient clinic visits using CPT codes 99201–99205 (new patient visits) and codes 99211–99215 (return patient visits). We used National Provider Identifier (NPI) numbers to identify the physicians and providers for each of these visits. We included both individual physician NPIs and organizational (i.e., physician group practice) NPIs. We used IQVIA data to identify individual physicians practicing within each organizational NPI. We excluded individual NPIs of advanced practice providers (e.g., nurse practitioners and physician assistants) since Colorado law does not allow these practitioners to prescribe MAID.

### Survey methods

We sent three waves of surveys (200 surveys in each of the first two waves, 183 surveys in the third wave; 583 total surveys). For each survey wave, we ranked physicians in order of what we believed to be their likelihood of being asked about MAID (stratified probability sample) based on the experience of the research team and responses from the first and second survey waves. A physician could be surveyed only once; if they were surveyed in a prior wave, they were excluded from the ranking process in subsequent waves.

For the first wave, ranking criteria consisted of two elements: patient to provider ratio, and practice specialty. For patient to provider ratio, we assigned points to physicians and organizations based on the number of patients in our cohort they had seen (e.g., an individual physician who saw 2 patients in the cohort had a ratio of 2, and an organization with 5 physicians and saw 10 patients also had a ratio of 2). Physicians or organizations with a patient to provider ratio of > 3 received 3 points; 2–3 received 2 points; 1 received 1 point; and < 1 received 0 points. Physicians who practiced within the same organization received the same number of points for this criterion, since organizational NPIs could not be assigned to individual physicians practicing under the organizational NPI. For practice specialty, medical hematology/oncology, hospice and palliative medicine, and geriatric medicine received 3 points; neurology, family medicine, and internal medicine received 2 points; pulmonary and cardiovascular medicine received 1 point; and all others received 0 points. This was based on descriptive reports from the CDPHE on the diagnoses of patients who were prescribed MAID. We then surveyed the 100 highest ranked physicians in each of the individual and organizational NPI groups (n = 200 total). Surveys were sent in two color codes, one for physicians with individual NPIs and one for physicians with organizational NPIs.

For the second wave, we varied the practice specialty ranking criteria and added a ranking criterion based on physician practice setting. This was based on results from the first wave suggesting that the vast majority of physicians who participated in MAID activities provided care in both the inpatient and outpatient setting, rather than just one setting. Specialists in medical hematology/oncology, hospice and palliative medicine, and geriatric medicine received 2 points; neurology, family medicine, and internal medicine received 1 point; and all others received 0 points. Physicians who billed in both the outpatient and inpatient settings received 1 point; those who billed in only one setting received 0 points. The patient to provider ratio ranking criteria were not changed. We then surveyed the 100 highest ranked physicians in each of the individual and organizational NPI groups (n = 200 total). As in wave 1, surveys were sent in two color codes based on use of individual or organizational NPI. For the third wave, we only surveyed those physicians who billed under their own individual NPI, and did not change any other ranking criteria from wave 2. This was based on results from the first and second wave suggesting that physicians who billed under their own individual NPI were more likely to have discussed MAID with their patients. All remaining physicians in the physician cohort with individual NPIs (n = 183) were surveyed in the third wave.

It is important to highlight how the survey administration differed from standard practice. First, given the sensitive nature of the topic we used a totally anonymous survey meaning there was no way to link responses to individual physicians. Second, we asked a very limited set of demographic questions with exceptionally broad response categories to further reassure respondents they could not be identified through their survey responses. Third, we utilized a very short survey (4 pages) that could be completed in under 15 min. Fourth, we provided an up front $50 cash incentive rather than a check or prepaid gift card as use of these mechanisms or incentives after completing the survey would necessitate identifying respondents to the research team. Finally, we did not do any follow-up activities such as telephone calls and additional mailings because we were not able to identify those who had responded to the survey and those who had not.

### Outcome measures

On the survey, we measured the extent to which physicians were prepared, willing, or had actually discussed MAID with patients, referred patients for MAID, served as a MAID consultant, and served as a MAID attending. Because of the extreme level of sensitivity around MAID and to assure physicians of their absolute anonymity, we asked a very limited set of demographic questions with intentionally broad response categories related to gender, specialty, length of medical practice, race/ethnicity and characteristics of their practice setting. Respondents were also instructed to feel free to skip any question they prefer not to answer.

### Statistical analysis

We used standard descriptive statistics (t-tests or Chi-square tests) to assess for differences in characteristics of clinicians surveyed and the self-reported characteristics of survey respondents. We used Chi-square tests to assess for differences in the proportions of survey respondents participating in MAID activities. Response rates were calculated according to the American Association for Public Opinion Research (AAPOR) standard definition version 4.1^12^. The study was approved by the Colorado Multiple Institutional Review Board. All research was performed in accordance with relevant guidelines/regulations. Since the survey was completely anonymous, the Colorado Multiple Institutional Review Board considered completion of the survey to be informed consent. The survey was conducted via traditional post. Participants were provided with an explanation of the study design and aims. Participants were also informed that survey results would be published.

### Ethics approval and consent

The study was approved by the Colorado Multiple Institutional Review Board.

## Results

### Characteristics of patients

**Figure 1:**
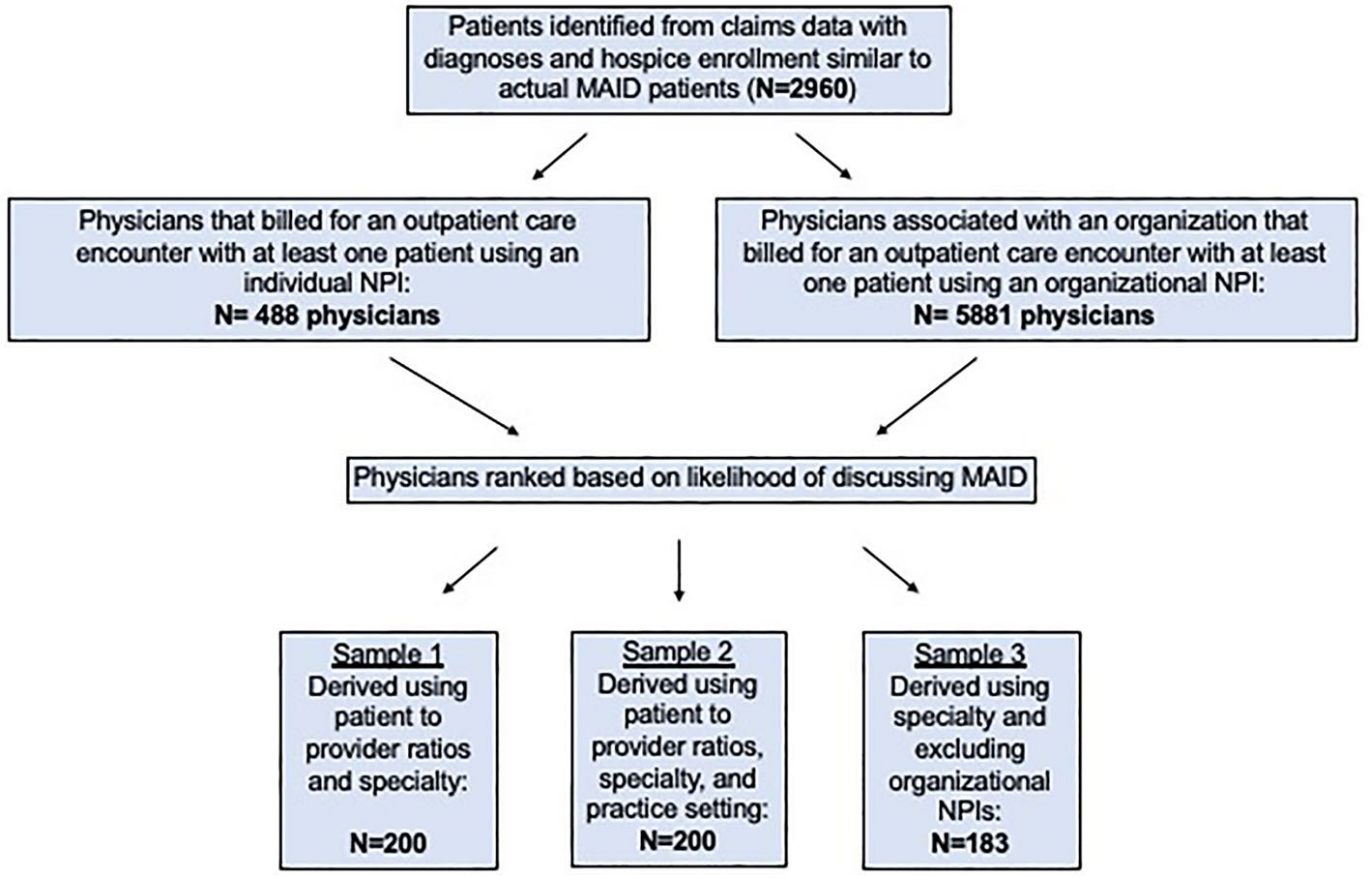
Flow diagram of survey sample creation. *MAID* medical aid-in-dying, *NPI* national provider identifier.

Study flow diagram is provided in Fig. 1. We identified 2960 patients (mean age 70, 54% women) with diagnosis codes matching those who received MAID prescriptions in Colorado and who received a hospice service (Table 1). Among these patients, 56% had malignant neoplasms, 53% had chronic pulmonary disease, 29% had heart failure, and 9% had a progressive neurologic disease (patients could have more than one diagnosis, thus percentages do not sum to 100).

**Table 1:** Characteristics of patients identified from administrative claims.

Total Patient Cohort (n)	2960
**Demographics**	
Age (mean, SD)	70 (21)
Women (n, %)	1614 (54%)
**Diagnosis**^**†**^ (n, %)	
Malignant neoplasms	1658 (56%)
Progressive neurologic disease	265 (9%)
Chronic pulmonary disease	1568 (53%)
Heart failure	846 (29%)

^**†**^Patients may be counted in more than one diagnosis group; thus percentages do not sum to 100. International Classification of Diseases, 10th Revision codes used are provided in the Supplemental Table.

### Characteristics of physicians

We identified 488 physicians that billed for an outpatient care encounter with at least one patient in our cohort using an individual NPI, and 5881 physicians associated with an organization that billed for an outpatient care encounter with at least one patient using an organizational NPI. These physicians were eligible for inclusion in our survey cohort, and were ranked according to the methodology described in the Methods section. Characteristics of physicians surveyed in each of the three waves is provided in Table 2.

**Table 2:** Characteristics of clinicians surveyed, N = 583.

	Sample 1 N = 200	Sample 2 N = 200	Sample 3 N = 183
**Women** (n, %)	99 (50%)	91 (46%)	57 (32%)
**Specialty** (n, %)			
Hematology and oncology	77 (39%)	27 (14%)	0
Hospice and palliative medicine	9 (5%)	4 (< 1%)	0
Geriatric medicine	3 (1%)	1 (< 1%)	0
Family medicine	37 (19%)	69 (35%)	48 (26%)
Internal medicine	63 (32%)	84 (42%)	58 (32%)
Neurology	11 (6%)	10 (5%)	6 (3%)
Pulmonary or cardiovascular medicine	0	5 (3%)	31 (17%)
Other^**†**^	0	0	40 (22%)
**Patient/provider ratio**			
1	43 (22%)	83 (42%)	177 (97%)
2–3	40 (20%)	16 (8%)	6 (3%)
> 3	117 (59%)	101 (51%)	0

^**†**^Other specialty includes: allergy & immunology, general surgery, infectious disease, obstetrics & gynecology, occupational medicine, pain medicine, psychiatry, sports medicine, surgical critical care, unspecified.

### Response rates and participation in MAID activities

**Figure 2:**
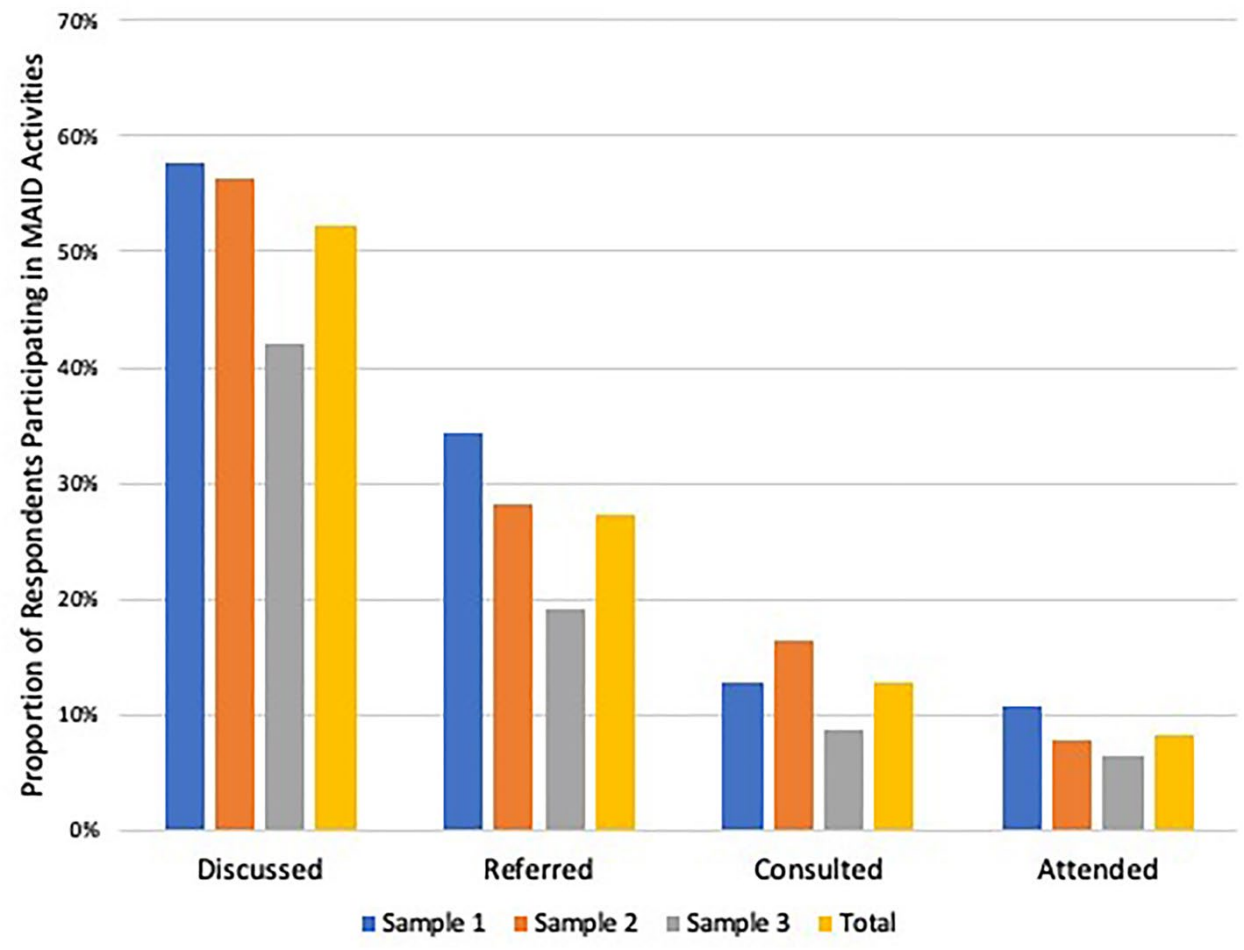
Proportion of Respondents Engaging in Any Medical Aid-in-Dying (MAID). Activity By Sample. Physicians who discussed MAID with patients, referred patient for MAID, consulted on a MAID patient, or attended (wrote a prescription) on a MAID patient in each survey wave.

We received 102 responses out of 185 eligible surveys in wave one (55.1% AAPOR adjusted response rate), 103 responses out of 188 eligible surveys in wave two (54.8% AAPOR adjusted response rate), and 95 responses out of 172 eligible surveys in wave three (55.2% AAPOR adjusted response rate; p = 0.98). Characteristics of respondents are provided in Table 3. The proportion of respondents that participated in any MAID activity (discussed, referred, consulted, or served as MAID attending with any patient) was 58%, 56%, and 42% in survey waves 1, 2, and 3 respectively (p = 0.05; Fig. 2). Across all three waves, the proportion of respondents who participated in MAID activities was 52%, and the proportion who participated as a MAID attending was 9%.

**Table 3:** Characteristics of Survey Respondents, N = 300.

	Sample 1	Sample 2	Sample 3	All
Total response, n	102	103	95	300
Women (n,%)	50 (49%)	43 (42%)	27 (28%)	120 (40%)
Non-white	25 (25%)	24 (23%)	17 (18%)	66 (22%)
**Specialty**				
Hematology/oncology	35 (36%)	17 (17%)	2 (2%)	54 (18%)
Palliative care	5 (5%)	3 (3%)	0	8 (3%)
Geriatrics	0	1 (1%)	0	1 (< 1%)
Family Medicine	19 (20%)	35 (34%)	31 (35%)	85 (28%)
Internal Medicine	28 (29%)	36 (35%)	16 (18%)	80 (27%)
Neurology	5 (5%)	5 (5%)	3 (3%)	13 (4%)
Pulmonary	0	1	3	4 (1%)
Cardiovascular	1	1	10	12 (4%)
Other	3 (3%)	4 (4%)	24 (27%)	31 (10%)
Missing	6	0	6	12
** > 20 Years in practice**	37 (36%)	52 (51%)	54 (57%)	143 (48%)
**Practice setting**^**†**^				
Inpatient	77 (75%)	45 (44%)	42 (44%)	164 (55%)
Outpatient	87 (85%)	92 (89%)	94 (99%)	273 (91%)
Nursing home	15 (15%)	14 (14%)	9 (9%)	38 (13%)
Hospice	5 (5%)	16 (16%)	7 (7%)	28 (9%)

^**†**^Categories for practice settings are not mutually exclusive, thus one could report multiple settings.

## Discussion

In this study, we report a novel methodology to identify and survey physicians likely to participate in MAID. We used administrative claims linked to a physician database to (1) identify a cohort of patients with characteristics similar to patients prescribed MAID in Colorado, (2) identify a cohort of physicians who provided routine outpatient care to these patients, and (3) survey these physicians in 3 separate iterative waves. We achieved good overall response rates (55%), and a high proportion of respondents that participated in MAID activities (52%).

Our study shows that it is possible to overcome two significant barriers to conducting quantitative research on MAID: survey cost and anonymity. The traditional way to study physicians’ experiences in MAID would be to follow the approach used by Ganzini et al., who in 1997 surveyed 4,500 physicians in Oregon in order to obtain responses from 122 physicians who reported writing a MAID prescription^3^. The cost of a $50 incentive alone for surveying 4500 physicians would be $225,000 alone. After accounting for the costs of survey development, administration, data entry, and analysis, the total cost of performing a survey using a similar method today is likely to exceed $2 million^7^. A second method would be to pre-screen 4,000–5,000 physicians by telephone or by mail and then follow-up with a survey for those who “screen positive” for MAID participation. This approach is still costly; moreover, it is not clear that this “hidden population” of physicians would be willing to answer screening questions about MAID and to do so truthfully when their responses to the screening questions could be directly linked back to them. Our approach, on the other hand, surveyed 545 eligible physicians to obtain responses from 283 physicians participating in MAID activities, incurred $300,000 in total costs, and was completely anonymous.

Illustrating the effectiveness of our targeting approach, the proportion of respondents who participated in MAID activities was 52%, and the proportion who participated as a MAID attending was 9%. This is in comparison to 0.01% of all Colorado physicians who are known to have participated as a MAID attending based on reports by the Colorado Department of Public Health and Environment. Our methodology therefore represents an efficient and anonymous alternative to the methods used in prior studies of physician experiences with MAID. Several unique methods likely contributed to our high proportion of MAID respondents. First, we targeted physicians providing care to terminally ill patients on hospice. Second, we employed methods to increase the probability that the physicians we surveyed discussed MAID with their patients, including ranking physicians based on 1) the number of unique patients in our cohort that they provided care to (i.e., the patient to provider ratio), and 2) based on specialty, with higher ranks going to specialties that we believed more likely to participate in MAID (e.g., oncologists and hospice and palliative medicine physicians). Third, our sampling strategy was iterative based on the results of prior survey waves; given the higher proportion of positive responses among physicians who billed using individual rather than organizational (i.e., group practice) NPIs, we surveyed only physicians that used individual NPIs in the third round.

To our knowledge, using administrative data to identify and survey a hidden population of physicians on highly sensitive research issues has not been previously reported in the literature. It is possible that similar approaches might be used to understand physician perceptions of other bioethics issues. For example, this methodology could be used to study doctors’ beliefs and behaviors regarding recommending marijuana in states in which marijuana use is illegal for medical or recreation uses.

There are likely ways in which the effectiveness and efficiency of our approach may be improved upon in future studies. First, our approach was designed such that physicians with the highest likelihood of participating in MAID activities (based on the a priori judgment of the research team) were targeted in the first round. The research team’s rankings proved successful enough that changes made to the rankings in rounds 2 and 3 did not increase the proportion of respondents that participated in MAID activities. Subsequent studies could consider using just one targeted survey wave. Second, mortality data were not available to us, but might be useful to identify physicians who cared for patients in the time before their death. Third, some of the cost savings from conducting a small targeted survey were offset by the costs of acquiring data from the APCD and IQVIA, but other ways to identify physicians likely to have participated in MAID activities without purchasing data could be explored. Fourth, since some states do not have all-payer claims data, using our methodology with similar data sources, such as Medicare claims, might be a viable approach.

Our study has several limitations. First, we used historical controls (costs and response rates from prior studies) rather than using a survey wave as a control. While this may limit some of our conclusions about costs and accuracy, we used this approach in order to iterate our sampling strategy based on results from prior waves. Second, we achieved similar response rates and proportions of physicians who participated in MAID activities in each survey wave. This may indicate the presence of a non-response bias. Third, the generalizability of our methods may be limited since we used data provided by the state of Colorado on patients who received MAID prescriptions, and data from the Colorado all-payer claims database. However, many other states where MAID is legal publish similar patient-level data and have developed all-payer claims databases. Fourth, our results are not generalizable to all physicians in Colorado as our samples were intentionally targeted towards the subpopulation of physicians caring for patients for whom MAID may be appropriate. Other physicians who participated in MAID activities could have been missed by our targeting method (i.e., possible selection bias).

## Conclusions

We used a novel methodology using administrative data to identify and survey physicians at high likelihood of participating in MAID activities. We achieved good overall response rates (55%), and a high proportion of respondents that participated in MAID activities (52%), demonstrating that it is possible to overcome survey cost and anonymity barriers to conducting quantitative research on MAID. This methodology could be used in larger scale studies of MAID or other bioethical issues with “hidden” physician populations.

## Supplementary Information

Supplementary Information.

## Data Availability

The Colorado All Payer Claims Database is available for purchase by researchers. Because of the sensitive nature of the data collected for this study, requests to access the dataset from qualified researchers trained in human subject confidentiality protocols may be sent to the Center for Improving Value in Healthcare at info@civhc.org. The survey dataset used in the current study is available from the corresponding author on reasonable request.

## Abbreviations

MAID: Medical aid-in-dying
APCD: All-payer claims database
NPI: National provider identifier
CDPHE: Colorado department of public health and environment
ICD-10: International classification of diseases, tenth revision
CPT: Current procedural technology
HCPCS: Healthcare common procedure coding system
AAPOR: American association for public opinion research
