# Supplementary material for: A novel methodology to identify and survey physicians participating in medical aid-in-dying
Source: Sci Rep. 2022 Apr 11;12:6056. doi: 10.1038/s41598-022-09971-7 (PMC9001750; doi:10.1038/s41598-022-09971-7)
Supplement: Supplementary file 1 — Supplementary Information. [file 41598_2022_9971_MOESM1_ESM.pdf]

SUPPLEMENTAL MATERIAL

Manuscript Title: A Novel Methodology to Identify and Survey Physicians Participating in Medical Aid-in-Dying

Authors: Vinay Kini, MD, MSHP, Bridget Mosley, MPH, Julie Ressalam, MPH, Dragana Bolcic-Jankovic, PhD, Hillary D. Lum, MD, PhD, Elizabeth R. Kessler, MD, Matthew DeCamp, MD, PhD, Eric G.Campbell, PhD

Supplemental Table: International Classification of Diseases, Tenth Revision (ICD-10), Current Procedural Technology (CPT), and Healthcare Common Procedure Coding System (HCPCS) Codes

Diagnosis	ICD-10	CPT	HCPCS
**Malignant neoplasms**	C34.XX, D02.20, D02.21, D02.22, Z85.110, Z85.118		
Lung	C34.XX, D02.20, D02.21, D02.22, Z85.110, Z85.118		
Pancreas	C25.XX		
Breast	C50.XXX, Z85.3		
Colorectal	C18.X, C19, C20, D01.0, D01.1, D01.2, Z85.038, Z85.040, Z85.048		
Head and Neck	C00.XX, C01.XX, C02.XX, C03.XX, C04.XX, C05.XX, C06.XX, C07.XX, C08.XX, C09.XX, C10.XX, C11.XX, C12.XX, C13.XX, C14.XX		
Central nervous system	C70.XX, C71.XX, C72.XX		
Esophagus	C15.XX		
Melanoma	C43.XX		
Ovary	C56.XX		
Kidney, bladder, and urinary tract	C64.XX, C65.XX, C66.XX, C67.XX, C68.XX		
Prostate	C61, D07.5, Z85.46		
Other	C22.0 (hepatocellular), C22.1 (cholangio), C91.X0 and C91.X2 (lymphoid leukemia not in relapse), C92.X0 and C91.X2 (myeloid leukemia not in replase), C93.X0 and C93.X2 (monocytic leukemia not in relapse), C90.X0 and C90.X2 (multiple myeloma not in relapse), C40.XX (bone and articular cartilage), C49.X (connective and soft tissue), C83.7 (Burkitt lymphoma), C83.3 (DLBCL), C84.A (cutaneous T cell lymphoma), C83.10 (mantle cell lymphoma)		
**Progressive Neurologic Disorder**			
Amyotrophic lateral sclerosis	G12.21		
Progressive supranuclear palsy	G12.22		
Parkinson’s disease	G20.XX		
Other	G35 (multiple sclerosis)		
**Chronic respiratory disease**	J40, J41.0, J41.1, J41.8, J42, J43.0, J43.1, J43.2, J43.8, J43.9, J44.0, J44.1, J44.9, J47.0, J47.1, J47.9		
**Heart failure**	I09.81, I11.0, I13.0, I13.2, I50.XXX		
**Hospice services**		99377 and 99378: bill for care	G0182: modifier for care planning for a patient on hospice

		planning for a patient on hospice<br><br>Modifiers GW or GV on any outpatient visit (99201-99205 for NPV, 99211-99215 for RPV) indicate that the patient is on hospice	G0337: hospice evaluation and counseling services, pre-election<br><br>G9473-9479: home services performed by non-physician staff (therapy, chaplain, etc) for a patient on hospice<br><br>Q5004-Q5010: hospice care provided in a facility<br><br>S0255: hospice referral visit performed by RN, SW, or other staff<br><br>T2042-T2046: hospice care per diem
